# Long-reads reveal that *Rhododendron delavayi* plastid genome contains extensive repeat sequences, and recombination exists among plastid genomes of photosynthetic Ericaceae

**DOI:** 10.7717/peerj.9048

**Published:** 2020-04-22

**Authors:** Huie Li, Qiqiang Guo, Qian Li, Lan Yang

**Affiliations:** 1 College of Agriculture, Guizhou University, Guiyang, Guizhou, China; 2 Institute for Forest Resources & Environment of Guizhou, Guizhou University, Guiyang, Guizhou, China

**Keywords:** *Rhododendron*, Ericaceae, Chloroplast genome, Repeat sequences, Gene recombination, Inverted repeat expansion, Phylogenetic relationship, Third-generation sequencing, De novo assembly, Chloroplast genome

## Abstract

**Background:**

*Rhododendron delavayi* Franch. var. delavayi is a wild ornamental plant species in Guizhou Province, China. The lack of its plastid genome information seriously hinders the further application and conservation of the valuable resource.

**Methods:**

The complete plastid genome of *R. delavayi* was assembled from long sequence reads. The genome was then characterized, and compared with those of other photosynthetic Ericaceae species.

**Results:**

The plastid genome of *R. delavayi* has a typical quadripartite structure, and a length of 202,169 bp. It contains a large number of repeat sequences and shows preference for codon usage. The comparative analysis revealed the irregular recombination of gene sets, including rearrangement and inversion, in the large single copy region. The extreme expansion of the inverted repeat region shortened the small single copy, and expanded the full length of the genome. In addition, consistent with traditional taxonomy, *R. delavayi* with nine other species of the same family were clustered into Ericaceae based on the homologous protein-coding sequences of the plastid genomes. Thus, the long-read assembly of the plastid genome of *R. delavayi* would provide basic information for the further study of the evolution, genetic diversity, and conservation of *R. delavayi* and its relatives.

## Introduction

The plant plastid genome is the genetic material that exists in the plastid independently of the nuclear genome. The genome has the characteristics of maternal inheritance in angiosperms, small genome size (usually about 120–160 k), and conserved gene number and structure (Dong et al., 2014). In recent years, a large number of plastid genomes have been revealed and compared for studying of taxonomy, evolution, breeding and conservation (Gruzdev et al., 2019a; Twyford & Ness, 2017; Zhong et al., 2019). The plastid genome of woody ornamental plant species such as *Prunus* (Cho, Yoon & Kim, 2018; Xue et al., 2019), *Rosa* (Jeon & Kim, 2019), *Paeonia* (Li, Guo & Zheng, 2018), *Camellia* (Li et al., 2019; Zhang et al., 2019), have also been established.

The development of the second-generation Illumina sequencing technology has led to the massive construction of plastid genomes at a low cost in the last decade. Although structural organization has been found to be highly conserved, there are exceptions. For example, plastid genomes have lost one copy of the inverted repeat (IR) in saguaro cactus (Sanderson et al., 2015), conifers (Hao et al., 2016; Ni et al., 2017) and some non-photosynthetic plants (Logacheva et al., 2016). Besides, drastic changes among plastid genomes such as rearrangement of genes, variation in gene number, expansion and contraction of IR region, repetition of fragments, have also increasingly reported (Zhang et al., 2018; Gruzdev et al., 2019b; Kang et al., 2019; Wang et al., 2020), which suggest the complexity of the plant plastid genomes. However, more recently, the long read lengths generated from third-generation sequencing technology have shown great advantages in the detection of structural variation and the analysis of highly variable regions. For instance, plastid genome of *Passiflora edulis*, the first assembled in Passifloraceae from long reads, differs from that of other species in Malpighiales because of rearrangement events (Cauz-Santos et al., 2017). Pacific Biosciences (PacBio) sequencing is the most commonly used third-generation sequencing technology generating long reads, its accuracy remains inferior to that of the second-generation Illumina sequencing technology (Tedersoo, Tooming-Klunderud & Anslan, 2018). Therefore, increasing attention has been paid to the combination of PacBio and Illumina techniques for genome assembly (Elbers et al., 2019).

*Rhododendron* belong to Ericaceae, has an important place in the gardening and landscape world. Abundant wild *Rhododendron delavayi* Franch. var. delavayi and its suspected natural hybridization and introgression offspring in Baili Rhododendron Reserve in Guizhou province, China, form a magnificent landscape of more than 120 square kilometers. The large red inflorescence of *R. delavayi* is highly attractive during the full bloom of spring. The natural in situ hybridization and introgression of this species with related species for a long period resulted in numbers of suspected offspring, which genetic background remains unclear. Although the draft genome of *R. delavayi* has been published (Zhang et al., 2017b), the lack of matrilineal genetic information seriously hinders further study on the application and conservation of these valuable ornamental resources. So far, plastid genomes of *Vaccinium macrocarpon* (Fajardo et al., 2013), *Arbutus unedo* (Martinez-Alberola et al., 2013) and *Pyrola rotundifolia* (Logacheva et al., 2016) of Ericaceae have been deciphered, but all of them are characterized by gene rearrangement, IR expansion, genome size variation and fragments repetition, indicating the complexity of the plastid genomes in Ericaceae.

Here we report the complete plastid genome of *R. delavayi* assembled on the basis of both PacBio and Illumina sequencing technology, and present a comparative analysis of this plastid genome and its photosynthetic Ericaceae relatives. This work will provide a valuable reference for further study on the evolution, breeding, and conservation of *R. delavayi*.

## Materials and Methods

### Sequencing and assembly

Fresh leaves of *R. delavayi* were sampled from Baili Rhododendron Nature Reserve of Guizhou, China. The herbarium voucher specimen for the sampled *R. delavayi* plant is held in Institute for Forest Resources and Environment of Guizhou, Guizhou University (20171012Rd, GACP), China. Total genomic DNA was extracted from leaves using a modified CTAB method (Healey et al., 2014), and then sent for sequencing. The 150-bp pair-end reads were generated by Illumina Hiseq platform (Illumina reads), and long reads were generated by PacBio Biosciences Sequel RSII platforms (PacBio reads) in Novogene, Inc, Beijing, China. The Illumina reads were trimmed, and then filtered with an average quality value less than Q5 or N content greater than five using fastp v0.20.0, https://github.com/OpenGene/fastp) to obtain the clean reads. The PacBio reads were corrected according to clean Illumina reads using LoRDEC v0.9 (Salmela & Rivals, 2014) with a *K* value of 22 and a solid threshold of 4. The corrected output reads were then checked with the lordec-trim command under default parameters, and were blasted against a plastid database in a local disk using minimap2 (2.2-r409) (Li, 2018). Next, hit reads above 1,000 bp were filtered by default and de novo assembled into contigs using Canu snapshot v1.5 with an overlap length greater than 800 bp. Subsequently, the longest contig was then identified as the primary plastid genome and polished with the clean Illumina reads using Pilon v1.22 (https://github.com/broadinstitute/pilon/) with default parameters. Finally, the borders between the quadripartite regions and the rearrangement and inversion of gene sets were verified by PCR and Sanger-sequencing technology. The PCR primers are listed in Table S1. Touchdown strategy of the annealing temperature was used in PCR to improve product specificity, which automatically reduced from 62 to 57 °C in 35 cycles. PCR products were first tested by agarose gel electrophoresis, the products with single band and expected length were sent for Sanger sequencing in Genewiz, Inc., Suzhou, China.

### Annotation

Coding sequence (CDS) annotation was performed by using Blast v2.2.25 (https://blast.ncbi.nlm.nih.gov/Blast.cgi) against the CDS database of plastid genomes downloaded from NCBI. Hmmer v3.1b2 (http://www.hmmer.org/) was used to compare the rRNA sequences of the genome against NCBI to obtain rRNA annotation, and Aragorn v1.2.38 (http://130.235.244.92/ARAGORN/) was used to predict tRNA and, finally obtain the tRNA annotation. OrganellarGenomeDRAW (OGDRAW) v1.3.1 (Greiner, Lehwark & Bock, 2019) was used to generate the plastid genome map.

### Repeat sequence analysis

MISA v1.0 (http://pgrc.ipk-gatersleben.de/misa/misa.html) was used for SSR and tandem repeat analysis. Parameters for SSR analysis were set as follows: eight for mononucleotides, five for dinucleotides, and three for trinucleotides, tetranucleotides, pentanucleotides and hexanucleotides. The minimum tandem repeat is set to seven, and the repetition number was set to two or more, including direct and palindromic repeats without mismatch. The palindromic repeats in the corresponding position of IRa and IRb were filtered. Vmatch v2.3.0 (http://www.vmatch.de/) was used for scattered repeats analysis. The minimum length was set to 15, and the repetition number was set to two or more.

### Comparison of plastid genomes among species

Comparative analysis were performed of IR border expansion or contraction among plastid genomes of photosynthetic Ericaceae. To show extensive recombination clearly, alignment was performed using Mauve v2.0 with default parameters (Darling, Mau & Perna, 2010) to detect gene rearrangement and inversion among plastid genomes of photosynthetic Ericaceae and of *Nicotinana tabacum*. Evolutionary analysis was conducted on the basis of 24 homologous CDSs which including *atpA*, *atpE*, *atpH*, *petG*, *petL*, *petN*, *psaA*, *psaB*, *psaC*, *psbB*, *psbC*, *psbD*, *psbE*, *psbF*, *psbH*, *psbI*, *psbJ*, *psbK*, *psbL*, *psbM*, *psbT*, *rbcL*, *rpl32* and *rps15*, of plastid genomes in 14 species, which including *R. delavayi*, nine other photosynthetic Ericaceae, three species from Theaceae of Ericales, and one species from Asteraceae of Asterales (outgroup). The collinear alignment of CDSs was carried out in Geneious R10 (Kearse et al., 2012), and the result was transferred into MEGA v6.0 (Tamura et al., 2013) to generate a maximum likelihood tree with 1,000 bootstrap replicates.

## Results

### Genome sequencing and assembly

**Figure 1:**
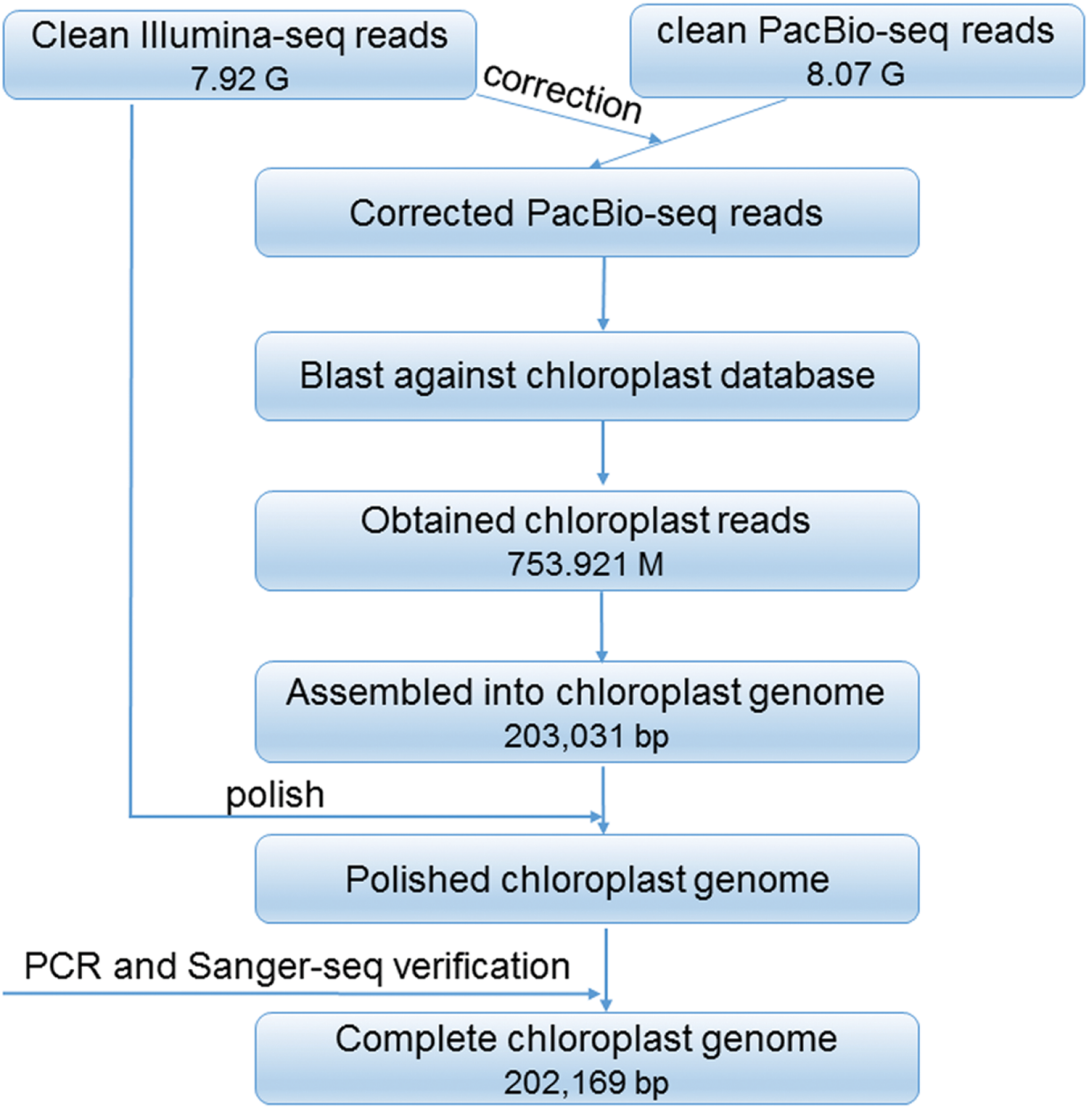
Cheme for the construction of the complete plastid genome of *R. delavayi*.

After trimming and filtering, 7.9 Gb clean reads sequenced by Illumina reads and 8.08 Gb reads sequenced by PacBio reads were generated. A total of 31 contigs were obtained. Among them, the longest one was 203,031 bp in length, which can be cyclized by overlap the two ends. This cyclized sequence has a quadripartite structure, and then recognized as the preliminary plastid genome of *R. delavayi*. The scheme for the construction of the complete plastid genome is showed in Fig. 1. In general, a total of 753.921 M PacBio reads were assembled, and the complete plastid genome has 971.85× mean coverage of PacBio reads, and 1,244.38× mean coverage of Illumina reads, and was deposited in GenBank under the accession number of MN711645.

### Genomic features

**Figure 2:**
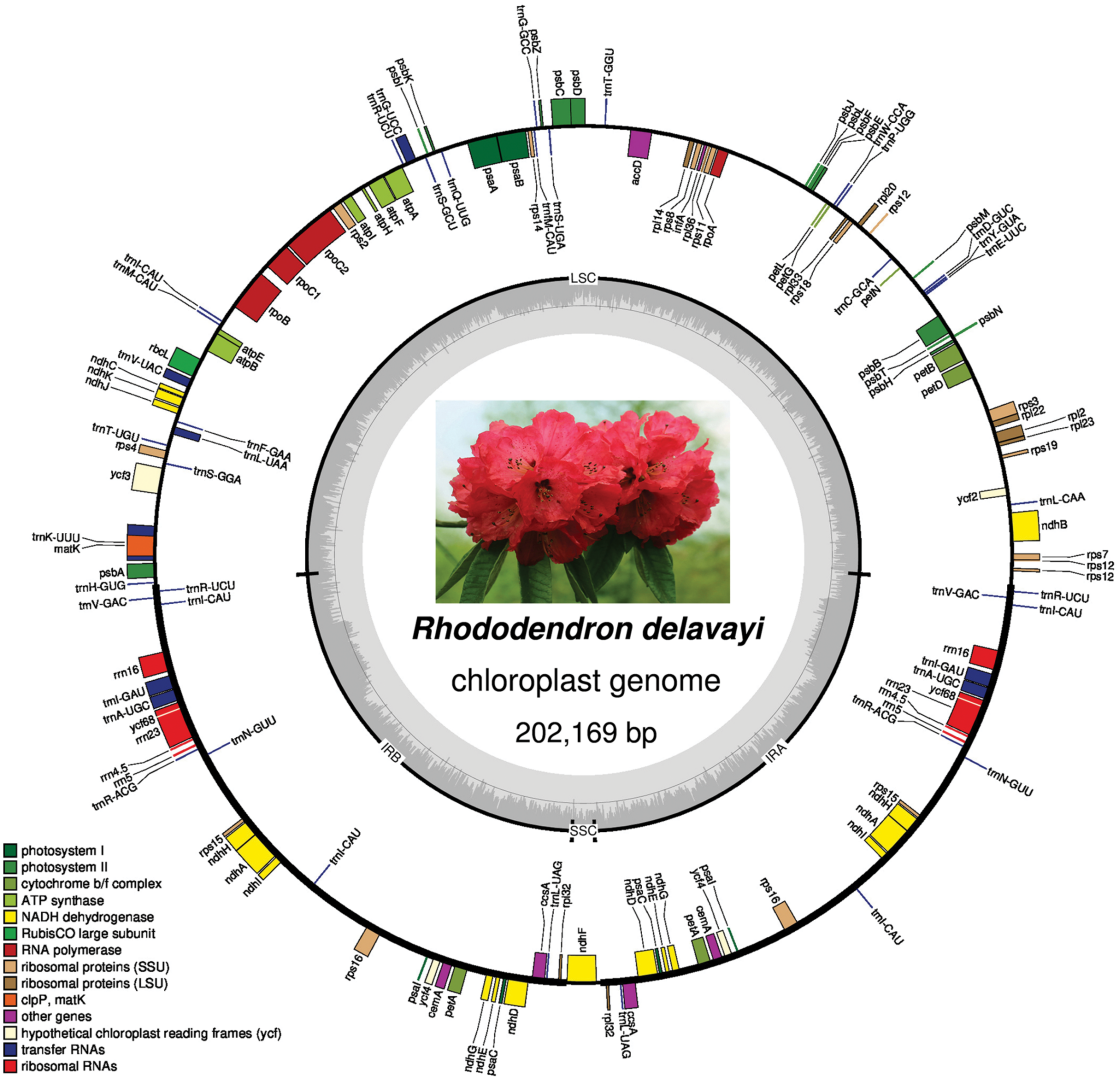
Plastid genome map of *R. delavayi*. The central part shows its blossom under natural conditions.

The plastid genome has a full length of 202,169 bp and contains a large single copy (LSC) with a length of 105,623 bp, two IRs with length of 46,968 bp each, and a small single copy (SSC) of 2,610 bp (Fig. 2). After annotation, a total of 137 genes were found in the plastid genome, including 88 protein-coding genes, 41 tRNAs, and 8 rRNAs. Although pseudogenes are common in the plastid genome of photosynthetic Ericaceae, only three pseudogenes were found in that of *R. delavayi*: *ycf2*, *rps19*, and *ycf68*, but *ycf1* was missing from plastid genome of *R. delavayi*.

### Codon usage and alternative initiation codons

Codon preference may be the result of natural selection, species mutation and genetic drift. Given the degeneracy of codons, each amino acid corresponds to at least one codon and at most six codons. Thus, the codon utilization rates of different species and organisms vary greatly. The inequality in the use of synonymous codons is called relative synonymous codon usage (RSCU). In this study, 20 amino acids are encoded by 65 codons (Fig. S1). The three most abundant amino acids are Leu, Ser and Arg. Six codons code for Leu (11.02%), among these codons, UUA has a RSCU value of 2.17, indicating that Leu is preferentially coded by UAA in *R. delavayi*. Six codons code for Ser (7.06%), among these codons, UCU has an RSCU value of 1.94, indicating that Ser is preferentially coded by UCU in *R. delavayi*. And 6 codons code for Arg (5.75%), among these codons, AGA has an RSCU value of 1.65, indicating that Arg is preferentially coded by AGA in *R. delavayi*. Conversely, Trp (1.83%) is coded by only TGG, and the absence of a preference result in an RSCU value of 1, which suggest that the Trp codon in *R. delavayi* is highly conserved. In most cases, AUG is used as the initiation codon for CDS, although RNA editing is sometimes observed in the initiation codon in some CDS of plastid genomes of certain plants (Raman & Park, 2016; Sablok et al., 2019; Wang et al., 2018). In this study, *ndhD* uses GUG as the initiation codon.

### Repeat sequences

The whole genome showed numerous repeated sequences, including tandem and scattered repeats (Table 1). Simple sequence repeat (SSR) analysis showed that the complete genome contains 371 SSRs in total, among which 233 are mononucleotide SSRs, with the most frequent repeats of A/T. Long repeat sequences throughout the genome are highly pronounced as well. For example, 96 tandem repeats were found in total, including a tandem repeat with length of 126 bp and two with a length of 505 bp. In addition, 1,868 scattered sequence repeats, including direct and palindromic repeats, were found in the genome. The lengths of 1,754 scattered repeats range from 15 bp to 100 bp and those 114 repeats range from 101 bp to 1000 bp. The detailed information of SSRs, tandem repeats and scattered repeats are listed in Tables S2, S3 and S4, respectively.

**Table 1:** Repeat sequence statistics of the complete plastid genome of *R. delavayi*.

Simple sequence repeats									
Unit length (nt)	1	2	3	4		5	6		Total
Number	233	16	101	18		2	1		371
Tandem repeats									
Unit length (nt)	7–10	11–20	21–30	52	126			505	Total
Number	66	17	8	2	1			2	96
Scattered repeats									
Unit length (nt)	15–100	101–200	201–300	301–400		401–600	601–1000		Total
Number	1,754	75	14	14		8	3		1,868

### Gene recombination

**Figure 3:**
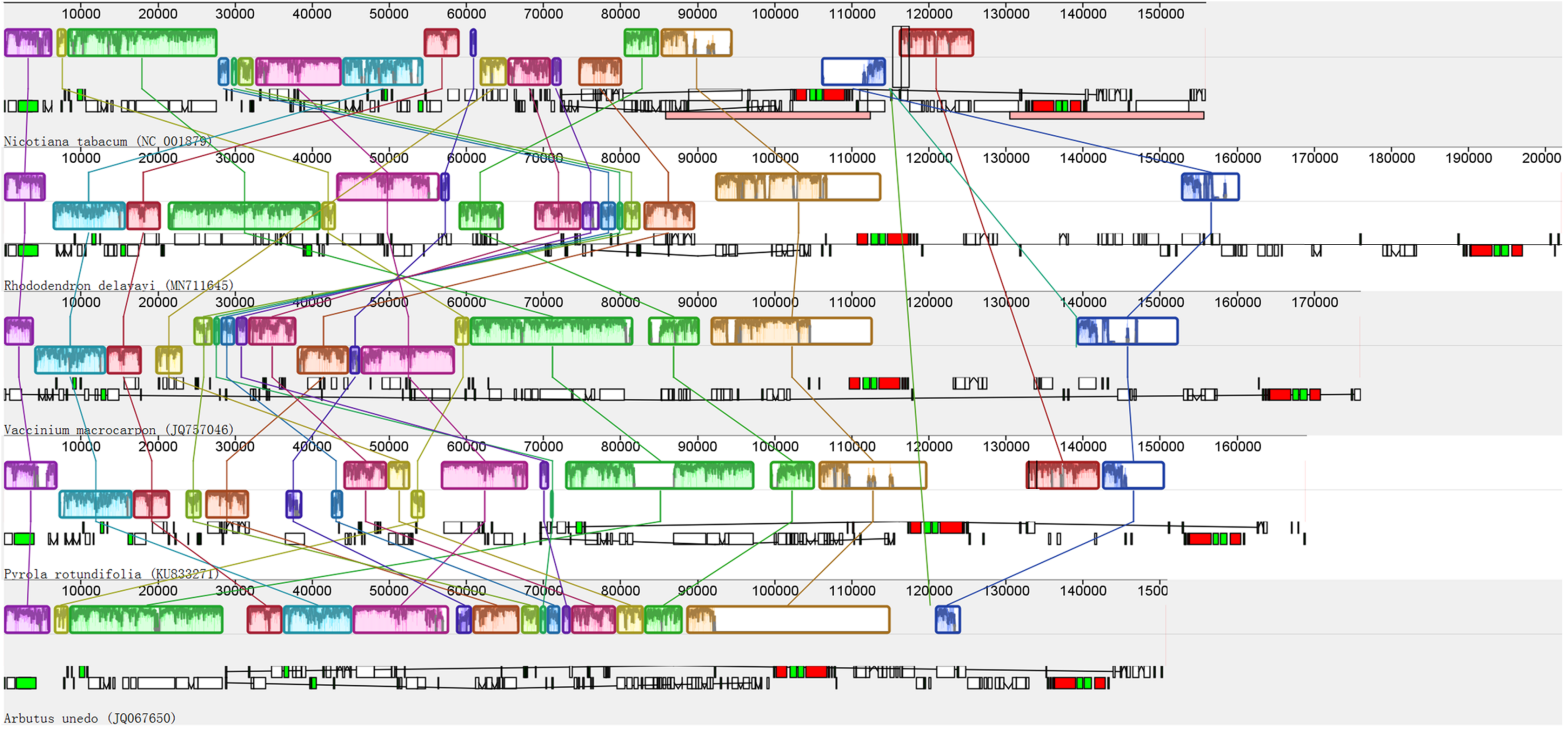
Alignment of the plastid genomes from four photosynthetic Ericaceae and *Nicotiana tabacum*.

Recombination, including rearrangement and inversion were detected in the de novo assembled plastid genome of photosynthetic Ericaceae. The results show that similar to most of the reported plant plastid genomes, the plastid genome of *R. delavayi* has a typical quadripartite structure; in contrast to those of photosynthetic Ericaceae, the genes of *R. delavayi* exhibit extensive recombination and inversion, particularly in the LSC region (Fig. 3). A comparison of the *R. delavayi* and *V. macrocarpon* from the same family (Fajardo et al., 2013), revealed that three gene sets are rearranged and inverted in the LSC of *V. macrocarpon*. The first set of *R. delavayi* includes 25 genes: *rpoB*, *rpoC1*, *rpoC2*, *rps2*, *atpI*, *atpH*, *atpF*, *atpA*, *trnR-UCU*, *trnG-UCC*, *trnS-GCU*, *psbI*, *psbK*, *trnQ-UUG*, *psaA*, *psaB*, *rps14*, *trnfM-CAU*, *trnG-GCC*, *psbZ*, *trnS-UGA*, *psbC*, *psbD*, *trnT-GGU* and *accD* (boxes three to six of *R. delavayi* in Fig. 3). The second set includes six genes: *rpl14*, *rps8*, *infA*, *rpl36*, *rps11* and *rpoA* (boxes seven to eight of *R. delavayi* in Fig. 3). The third set includes 16 genes: *psbJ*, *psbL*, *psbF*, *psbE*, *petL*, *petG*, *trnW-CCA*, *trnP-UGG*, *rpl33*, *rps18*, *rpl20*, *trnC-GCA*, *petN*, *psbM*, *trnD-GUC*, *trnY-GUA* and *trnE-UUC* (boxes nine to thirteen of *R. delavayi* in Fig. 3). Besides, this rearrangement and inversion occurred not only between these two related species, but also among *Nicotinana tabacum* and other photosynthetic Ericaceae (Fig. 3). Therefore, in contrast to the conserved plastid genomes of most other photosynthetic angiosperms, the four plastid genomes of photosynthetic Ericaceae show extensive irregular rearrangement and inversion, suggests that each plastid genome of photosynthetic Ericaceae has their own specific characteristics.

### IR expansion or contraction

**Figure 4:**
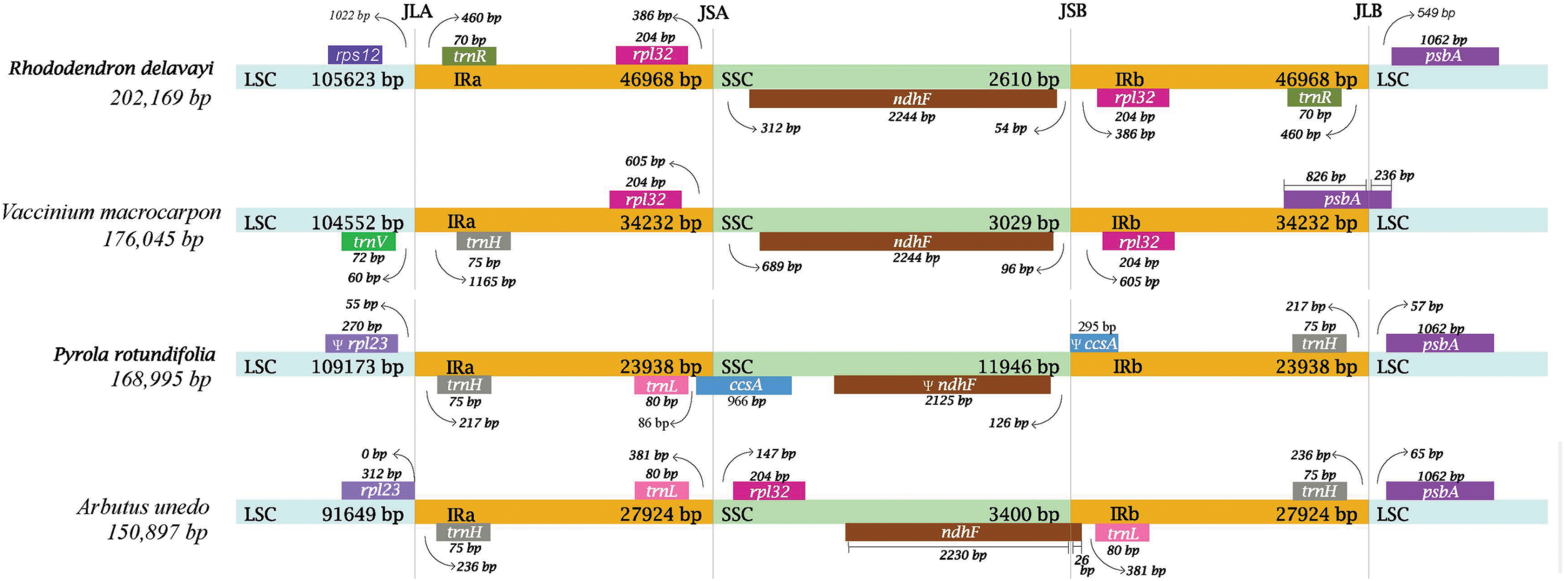
Comparison of the borders of the LSC, SSC and IR regions of four plastid genomes from photosynthetic Ericaceae.

In Ericaceae plants, IR of *R. delavayi* was significantly expanded with a length of 46,968 bp, followed by that of *V. macrocarpon* with a length of 34,232 bp, *A. unedo* with a length of 27,942bp and *P. rotundifolia* with a length of 23,938 bp (Fig. 4). In the plastid genome of *R. delavayi*, on one hand, some of the genes normally found in the LSC of conserved plastid genome, like *rps16*, *psaI*, *ycf4*, *petA* and *cemA*, have moved to the IR of *R. delavayi*, and other genes normally found in SSC, like, *nhhA*, *ndhD*, *ndhE*, *ndhG, ndhH*, *ndhI*, *rps15*, *psaC*, *ccsA*, *rpl32*, also have moved to the IR, on the other hand, the genes, like *rpl2*, *rpl23*, *ycf2*, *ndhB*, *rps7* and two exons of *rps12*, have moved from IR to LSC. In general, more genes moved into IR than out of IR in plastid genome of *R. delavayi*, which eventually led to an increase in its complete length.

### Phylogenetic relationship

**Figure 5:**
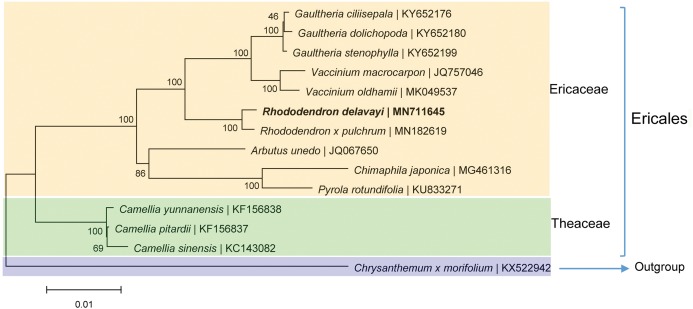
Phylogenetic trees based on homologous CDSs of chloroplast genomes from photosynthetic Ericaceae.

The phylogenetic analysis based on the homologous CDSs showed that *R. delavayi* is most closely related to *R. pulchrum* with 100% support, these two *Rhododendron* with eight other species of the same family were clustered together (Fig. 5). Theaceaebelonged to Ericales clustered relatively close to Ericaceae (Fig. 5), indicating that the topology is consistent with traditional taxonomy.

## Discussion

Ericaceae includes photosynthetic and non-photosynthetic plants, non-photosynthetic Ericaceae species are usually chlorophyl-deficient and mycoheterotrophic. The plastid genomes of Ericaceae plants vary greatly in length, for example, the plastid genomes of a non-photosynthetic species *Monotropa uniflora* has a length of only 45,111 bp, that of another non-photosynthetic species *Hypopitys monotropa* is only 35,062 bp. In both plants, the gene set is greatly reduced because all the genes encoding the components of the photosynthetic apparatus are missing or pseudogenized, resulting in small genome size (Logacheva et al., 2016). By contrast, the plastid genomes of photosynthetic Ericaceae are usually long: they are 150,897 bp for *A. unedo* (Martinez-Alberola et al., 2013), 168,995 bp for *P. rotundifolia* (Logacheva et al., 2016), 176,045 bp for *Vaccinium macrocarpon* (Fajardo et al., 2013), and 202, 169 bp for *R. delavayi* in this study. However, most of the reported conserved plastid genomes are usually 120–160 kb in length (Orton, Burke & Duvall, 2019; Twyford & Ness, 2017; Xue et al., 2019). In addition, *ycf1*, which is common in conserved plastid genomes, is missing from *R. delavayi*, and from other photosynthetic Ericaceae (Kim et al., 2019; Logacheva et al., 2016; Martinez-Alberola et al., 2013).

Plastid sequences usually have a lower repeat rate than nuclear and mitochondrial genomes, especially for long repeats, and most of the short repeats are mononucleotide A/T (Marechal & Brisson, 2010; Twyford & Ness, 2017). For example, A/T is the most abundant among all SSR in the plastid genomes of *Camellia*, but only 156 repeats exceed 30 bp in length, and the longest repeat is 82 bp in length (Huang et al., 2014), which is shorter than the reads generated by most next-generation sequencing technologies. However, in this study, except for the fact that the A/T mononucleotide is the most abundant SSR, the plastid genome of *R. delavayi* contains a large number of repeat sequences, and many long repeats, including tandem and scattered repeats, have lengths of more than 150 bp. Similarly, Logacheva et al. (2016) have pointed that five plastid genomes of Ericaceae also contain numbers of repeats that differ among sequences regardless of their photosynthetic capacity, indicating that all the reported plastid genomes of Ericaceae contain abundant repeats.

The existence of a large number of repeat sequences is regarded to be the basis of genome homologous recombination and plastid genome rearrangements and inversion, and the number and distribution of these repeats vary from one species to another (Cauz-Santos et al., 2017). In this study, the significant recombination of gene sets were found among plastid genomes of *R. delavayi* and the related species, which not only resulted in changes in gene order, but also in gene diversity. For example, two exons in the *rps12* gene of *R. delavayi* are separated by IR and SSC, the same phenomenon was observed in *A. unedo* (Martinez-Alberola et al., 2013), while only one trans-spliced *rps12* was found in *P. rotundifolia* (Logacheva et al., 2016) and only pseudogenes of *rps12* were retained in *V. macrocarpon* (Fajardo et al., 2013). In addition, recombination was also found among plastid genomes of Geraniaceae (Guisinger et al., 2011), Cupressophytes and Pinaceae (Wu et al., 2011; Hao et al., 2016), Passifloraceae (Cauz-Santos et al., 2017), Caryophyllaceae (Kang, Lee & Kwak, 2017), Apiaceae (Lee et al., 2019).

The IR and SSC regions of plant plastid genomes are more conserved and less prone to recombination than the LSC region (Fajardo et al., 2013). However, the expansion of the IR from single copy region often change the lengths of the IR, which is usually 20–25 kb in length of conserved plastid genomes. In this study, the length of IR of *R. delavayi* is almost twice that of the conserved, and is also the longest one among the reported plastid genomes from photosynthetic Ericaceae. Though expansion and contraction of IR region are common in plant plastid genome, they are in different degree. For example, the extreme shortening of the SSC was due to the expansion of the two IR, which expanded to 34,232 bp for each in *V. macrocarpon* but not in *A. unedo* (Fajardo et al., 2013; Martinez-Alberola et al., 2013). In this study, the IR expansion is more prominent in *R. delavayi* compared with that of *V. macrocarpon* (Fajardo et al., 2013). This prominent IR expansion, combined with numerous repeats in plastid genome of *R. delavayi* may be the main reasons that resulted in its long plastid genome of.

It’s worth noting that the recent published plastid genome of *R. pulchrum* assembled from Illumina reads shows that the length of the plastid genome *R. pulchrum* is only 136,249 bp, and have lost one of the IR copy (Shen et al., 2019). Though phylogenetic analysis showed that *R. delavayi* is most closely related to *R. pulchrum* with 100% support, these two plastid genomes are still very different in length. Since the assembly information of *R. delavayi* based on second-generation sequencing technology are very limited, it is still unclear whether or not the big variations in length and structure between the two *Rhododendron* plastid genomes can be attributed to species differences. Similarly, the plastid genomes of *Gaultheria* of Ericaceae also varied widely in length, which are ranged from 62,505 bp to 163,526 bp in length (Zhang et al., 2017a). These further suggests the complexity of plastid genomes from Ericaceae.

By now, although a few plastid genomes of Ericaceae are published, species specificity remains striking. The complete plastid genome of one species in photosynthetic Ericaceae is likely of little reference value for another. Given that the advantages of long-read sequencing technology, the plastid genome of *R. delavayi* could be assembled in this study. In the future, with the development of technology, it is hoped that more plastid genomes of Ericaceae will be deciphered to reveal more characteristics.

## Conclusions

In this study, the complete plastid genome of *R. delavayi* was de novo assembled from long reads. It is 202,169 bp in length and contains a LSC of 105,623 bp, two IRs of 46,968 bp each, and a SSC of 2,610 bp. In addition, the complete genome contains a large number of repeated sequences and shows codon preference. The comparative analysis of the plastid genomes in photosynthetic Ericaceae showed that gene sets have been irregularly rearranged in LSC region, and the prominent expansion of IR regions. Consistent with traditional taxonomy, *R. delavayi* with nine other species of the same family were clustered together based on the homologous CDSs of the plastid genomes. The long-read assembly of the plastid genome of *R. delavayi* may provide insight into other uncovered plastid genomes, and also provide basic information for further studies on the evolution, hybrid introgression, parental identification and genetic diversity and conservation of *R. delavayi* and its relatives.

## Supplemental Information

10.7717/peerj.9048/supp-1 Supplemental Information 1 Relative Synonymous Codon Usage.

10.7717/peerj.9048/supp-2 Supplemental Information 2 Primers information of PCR verification.

10.7717/peerj.9048/supp-3 Supplemental Information 3 Details of SSRs.

10.7717/peerj.9048/supp-4 Supplemental Information 4 Details of scattered repeats.

10.7717/peerj.9048/supp-5 Supplemental Information 5 Details of tandem repeats.

## Additional Information and Declarations

### Field Study Permissions

The following information was supplied relating to field study approvals (i.e., approving body and any reference numbers): Because the samples were collected from only a few leaves of a large trees, which had no effect on the growth of the trees, no permission was required.

### DNA Deposition

The following information was supplied regarding the deposition of DNA sequences: Data is available at GenBank: MN711645.

### Data Availability

The following information was supplied regarding data availability: Raw data is available at the SRA database: PRJNA596002. The herbarium voucher specimen (20171012Rd) for the sampled *R. delavayi* plant is held in Institute for Forest Resources & Environment of Guizhou, Guizhou University, China.
