# Supplementary figures and images for: Long-reads reveal that Rhododendron delavayi plastid genome contains extensive repeat sequences, and recombination exists among plastid genomes of photosynthetic Ericaceae
Source: PeerJ. 2020 Apr 22;8:e9048. doi: 10.7717/peerj.9048 (PMC7183307; doi:10.7717/peerj.9048)

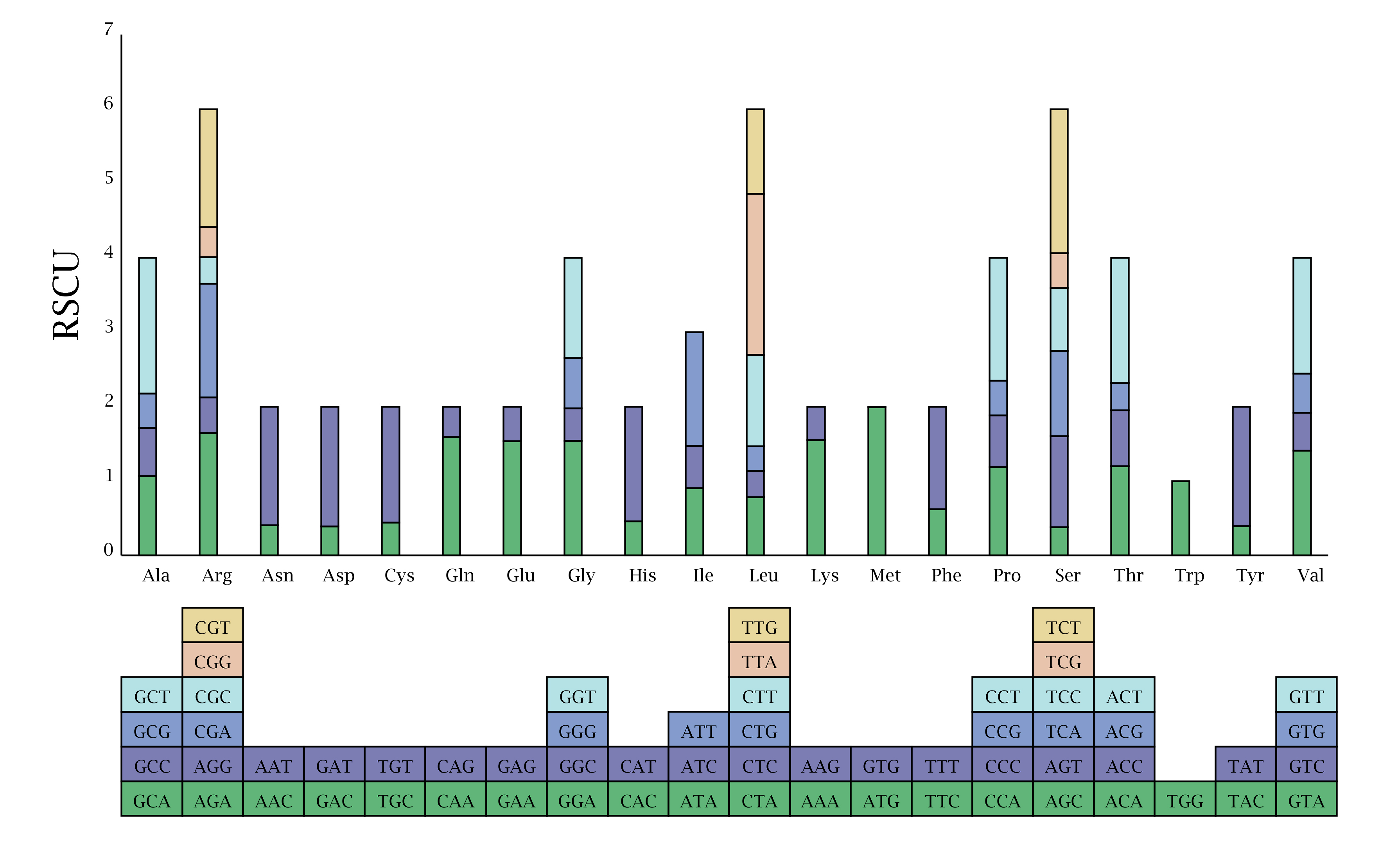

Supplement: Supplemental Information 1 [file peerj-08-9048-s001.jpg]
